# Caecal Volvulus in a Foramen of Winslow Hernia Masquerading as Biliary Colic

**DOI:** 10.7759/cureus.66523

**Published:** 2024-08-09

**Authors:** Ziad Zeidan, Mohamed Aburrous, Parag Dhumane, Sivakumar Gopalswamy, Ponnandai Arumugam

**Affiliations:** 1 Department of Emergency Abdominal Surgery, Royal Cornwall Hospital Trust, Truro, GBR

**Keywords:** diagnostic momentum, cholelithiasis, computed tomography, foramen of winslow, internal hernia

## Abstract

Internal hernias account for a minority of cases of intestinal obstruction. Within this group, internal hernias through the foramen of Winslow (FW) are an even rarer subcategory with a paucity of cases reported in the literature. We present a case of a 48-year-old female presenting with right upper quadrant pain akin to biliary colic with sonographic evidence of cholelithiasis. Her symptoms swiftly worsened, and she re-presented with symptoms of bowel obstruction. She was subsequently found to have a caecal volvulus herniating through the FW on computed tomography (CT). She underwent an emergency laparotomy to reduce the hernia and prevent further recurrence, which highlighted the importance of a comprehensive history and the increasing role of cross-sectional imaging in emergency surgery.

## Introduction

The foramen of Winslow (FW) is a small window that connects the greater peritoneal cavity and the lesser sac [1]. Under normal circumstances, it is closed due to intrabdominal pressure exerted by the organs in its surroundings [1]; anteriorly, it is bordered by the hepatoduodenal ligament [1,2]. Internal hernias, meanwhile, account for up to 5.8% of all causes of intestinal obstruction, with an incidence of 1% [1]. Furthermore, they have a mortality of 50%, partly explained by the non-specific symptoms attributed to them and subsequent delays in diagnosis [1]. Overall, the incidence of internal hernias is increasing, particularly with the increasing frequency of bariatric surgery in practice, which leads to rapid loss of visceral fat [1]. Within this subgroup, FW hernias account for only 8% of internal hernias, with 150 cases reported in a literature review from 2019 [1-3]. Furthermore, eight cases have been identified reporting a caecal volvulus within the lesser sac, as demonstrated in our patient [3,4]. The aim of this case report is to highlight the diagnostic challenges these cases present and the challenges of managing them surgically. 

## Case presentation

**Figure 1:**
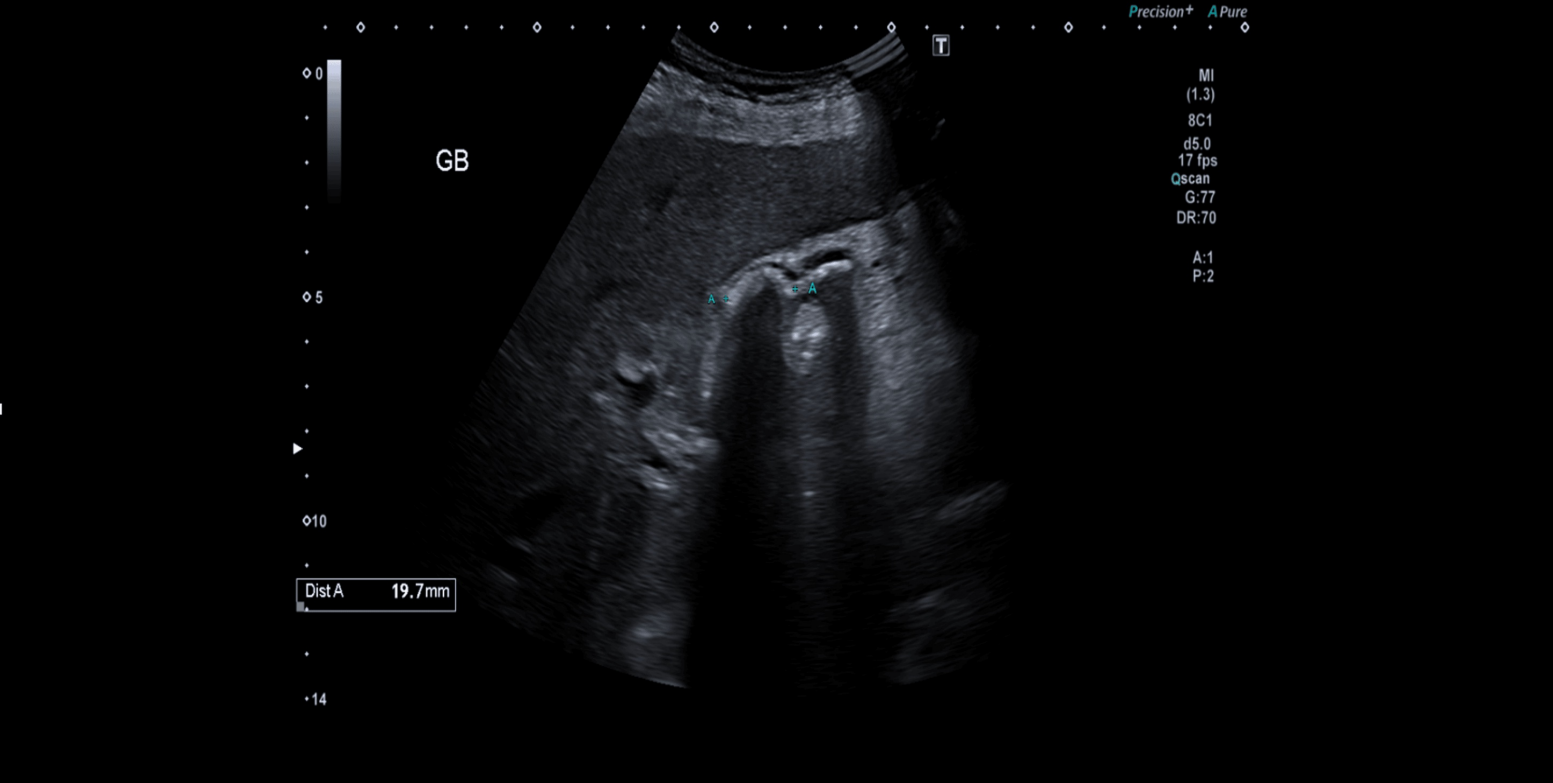
Abdominal ultrasound image of the patient on initial presentation demonstrating a thin-walled gallbladder without evidence of cholecystitis.

A 48-year-old female with multiple comorbidities was referred to our emergency surgical assessment unit with recurrent right upper quadrant pain and nausea after meals suggestive of biliary colic. Significantly, she was diagnosed with antiphospholipid syndrome in 2021, after developing a pulmonary embolism in the same year, for which she was receiving oral rivaroxaban. Her other medical comorbidities included ischaemic heart disease, having sustained a myocardial infarction in 2020, along with at least a 30-pack-year smoking history. Her symptoms had been ongoing for around four months and were attributed to cholelithiasis, which was diagnosed in another hospital. She was haemodynamically stable and apyrexial on initial presentation. On examination, she had a soft, non-peritonitic, and non-distended abdomen with tenderness confined to the epigastrium and right upper quadrant. In addition, Murphy’s sign was negative. An inpatient ultrasound (USS) demonstrated two 2 cm gallstones in a thin-walled gallbladder (Figure 1) alongside completely normal blood results, including liver function tests (LFTs) (Table 1). Her symptoms were, therefore, attributed to being due to cholelithiasis. Given these reassuring findings, her pain relief was optimised with oral paracetamol and morphine, and she was discharged the following morning after being added to the waiting list for a laparoscopic cholecystectomy within the next two weeks in view of her ongoing symptoms. 

**Table 1:** Table comparing blood results from the patient on initial admission and second admission with the significant finding of deranged liver function tests (LFTs) in keeping with possible biliary obstruction

	Initial admission	Second admission	Reference range
White cell count	8.9x10^9^/L	8.0x10^9^/L	3.9-11.1x10^9^/L
C-reactive protein	1 mg/L	12 mg/L	0-5 mg/L
Bilirubin	6 micromol/L	30 micromol/L	0-21 micromol/L
Alkaline phosphatase (ALP)	80 iu/L	336 iu/L	30-130 iu/L
Alanine transaminase (ALT)	13 iu/L	440 iu/L	0-55 iu/L

The patient presented again after two days to our emergency surgical assessment unit with worsening pain and vomiting. She was still haemodynamically stable and apyrexial, with largely similar examination findings. Her blood tests were significant for a bilirubin of 30 micromol/L, alanine transaminase (ALT) of 440 IU/L, and alkaline phosphatase (ALP) of 336 IU/L (Table 1). Given her previous attendance, choledocholithiasis was considered the primary differential diagnosis, and she underwent an urgent magnetic resonance cholangiopancreatography (MRCP). This demonstrated what appeared to be a closed-loop bowel obstruction in the right upper quadrant with no obstructing calculi in the common bile duct (CBD). The radiologist noted tapering of the extrahepatic duct at its midpoint, in keeping with external compression; the two gallbladder calculi were also noted, with no evidence of cholecystitis. This finding was urgently communicated to the surgical team, who revisited the history of the patient.

**Figure 2:**
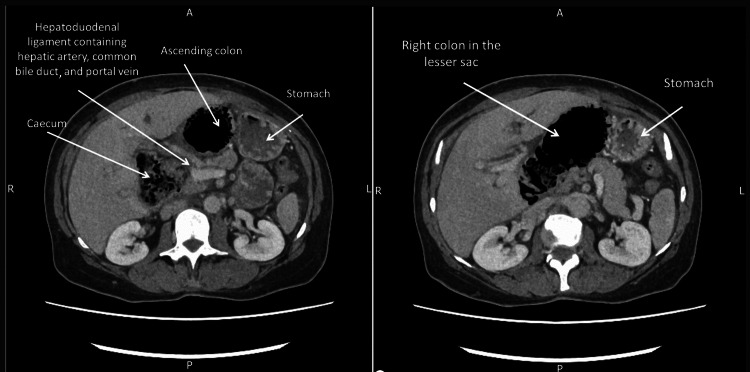
Axial CT scan images showing the right colon in the lesser sac with pneumatosis intestinalis suggesting ischaemia (right) and demonstrating the herniated content behind the hepatoduodenal ligament (left).

**Figure 3:**
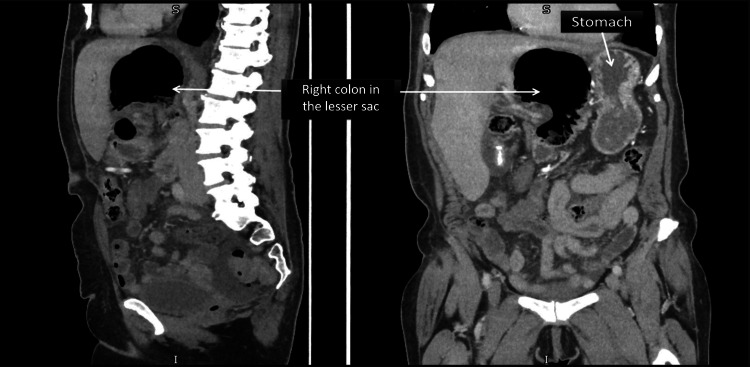
Sagittal (left) and coronal (right) CT views demonstrating the right colon within the lesser sac

It transpired that she had not passed stool in three days, and in conjunction with a 48-hour history of persistent nausea and vomiting after food, the diagnosis of an internal hernia causing intestinal obstruction became evident. She underwent an urgent computed tomography (CT) scan of the abdomen and pelvis that was urgently reported by a gastrointestinal radiologist. The scan demonstrated that a volved caecum was herniating through the FW with a closed loop large bowel obstruction. Furthermore, the dilated large bowel was causing external compression of both the portal vein and the common bile duct (Figures 2-3). Significantly, the radiologist reported pneumatosis intestinalis, which was concerning for bowel wall ischaemia (Figure 2). In addition, the CT was reported to demonstrate superior mesenteric vein thrombosis. 

It became apparent that this finding may have been masquerading as biliary colic for three days, and the decision to undertake an urgent laparotomy was made. 

Our patient was taken to the operating theatre and the CT findings were confirmed upon entering the peritoneal cavity. The liver initially appeared to be congested and dusky. Meanwhile, the FW was carefully widened, taking into consideration the neighbouring hepatoduodenal ligament, and the herniated volved caecum was identified in the clockwise orientation. This was detorted with gentle manipulation in the opposite direction to the initial orientation. Caution was taken to avoid injuring the splenic artery at this point as well, which was posterior to the hernia. The herniated caecum initially appeared dusky, but following reduction, it returned to a normal pink colour with no serosal injury identified. Obliteration of the FW was achieved with 3-0 polypropylene non-absorbable sutures. In addition, a caecopexy was undertaken to the lateral abdominal wall with 3-0 polypropylene sutures to prevent further recurrence. This approach was preferred to undertaking a right hemicolectomy in view of the healthy tissue identified intraoperatively, with the risk of anastomotic leak being significant given the patient's medical comorbidities. Following this, the patient was commenced on a heparin infusion in view of her mesenteric thrombosis and two days later switched to oral rivaroxaban. 

## Discussion

As mentioned previously, cases of an internal hernia within the FW are extremely rare, which presents a challenge to diagnosis and surgical management. Cases of a caecal volvulus within the FW are a rare subset of this type of internal hernia, with a literature review in 2022 revealing eight cases [4]. In addition to this research, a PubMed search was carried out to identify articles in English with the keywords 'Foramen of Winslow', 'hernia', and 'obstructive jaundice', which identified seven articles describing FW hernias leading to biliary obstruction (Table 2).

**Table 2:** Comparison of case reports of foramen of Winslow (FW) hernias leading to biliary obstruction

Case number	Authors	Age	Sex	Region	Presenting complaint	Diagnostic modality and findings	Operative technique
Case 1	Joo et al. [5]	45 years	Male	South Korea	Epigastric pain and jaundice	CT: Dilated small bowel loops in gastrohepatic space with dilatation of gallbladder and both intrahepatic bile ducts	Laparotomy with manual reduction of herniated small bowel
Case 2	Antao et al. [6]	19 months	Sex not specified	United Kingdom	Jaundice	CT: Intestinal malrotation with extrinsic compression of the common bile duct (CBD)	Laparotomy with manual reduction of volvulus and hernia
Case 3	Welaratne et al. [7]	Middle-aged (exact age not reported)	Female	Republic of Ireland	Epigastric pain and jaundice	CT: Dilated caecum within lesser sac with mass effect on hepatic hilum	Laparotomy with manual reduction of herniated caecum followed by caecopexy
Case 4	Koh et al. [8]	53 years	Female	Australia	Right upper quadrant pain	Laparoscopy: Herniation of gallbladder into the lesser sac	Laparoscopic cholecystectomy
Case 5	Bozbulut et al. [9]	16 months	Female	Turkey	Abdominal pain and jaundice	CT: Herniation of ascending colon, caecum, and duodenum with compression of the CBD by duodenum	Laparotomy with manual reduction of hernia
Case 6	MacDonald et al. [10]	33 years	Female	United Kingdom	Abdominal pain and Jaundice	MRI: Herniation of caecum and ascending and transverse colon into the lesser sac with obstruction of the CBD	Laparotomy with manual reduction of hernia and surgical closure of the FW
Case 7	Tjandra et al. [11]	43 years	Female	Australia	Epigastric pain and jaundice	CT: Dilated intrahepatic bile ducts and gallbladder with mass lesion at porta hepatis	Laparotomy with manual reduction of the caecum and ascending colon along with a caecopexy to the right iliac fossa

From the perspective of first principles, the aetiology of this patient's hernia is unclear. In addition, given the gaps in her initial presenting history, it is unclear exactly how long she had this hernia. Nevertheless, what the clinicians involved could assume with reasonable evidence is she could have had this for three days, as this was when she last passed stool prior to presenting to the hospital the second time. Thereby, necessitating urgent surgical intervention. Furthermore, a mobile caecum would have certainly played a part in the development of this hernia, which is a finding present in 10%-20% of individuals [12]. It results from a failure of the right colonic mesentery to fuse with the lateral abdominal wall during embryogenesis, which results in a mobile caecum and ascending colon [9]. 

This case was also misleading to the clinicians involved due to the predetermined finding of cholelithiasis prior to her attending our unit. Further to this, following being discharged, she re-presented with worsening pain as her chief complaint, along with deranged LFTs suggestive of biliary obstruction. Given the acuity of these biochemical findings and the previous finding of gallstones, choledocolithiasis was the primary differential diagnosis considered. In spite of the patient’s constipation, intestinal obstruction was not considered primarily as she did not appear to have significant abdominal distension. When further information, in the form of the MRCP, became available against this diagnosis, the history was then revisited. It then became apparent that the patient had not passed stool in three days, and the indication for an urgent CT became clear. This demonstrates the phenomenon of diagnostic momentum in action, whereby clinicians become fixated on a predetermined diagnosis, in this case, cholelithiasis, diagnosed previously. 

Hence, this case highlights three key issues. Firstly, internal hernias are a diagnostic challenge due to the non-specificity of symptoms. In this case, the availability of rapid cross-sectional imaging with a reporting gastrointestinal radiologist assisted in establishing a diagnosis swiftly. In addition, an open mindset to other differentials is key and a prompt to revisit the history when a definitive diagnosis is not initially attained. In this case, the history was revisited when the investigations in the work-up for suspected choledocolithiasis did not yield the expected diagnosis. Finally, cases of an internal hernia are a challenge also from the perspective of surgical management. With a limited number of cases reported in the literature, experience or guidance in laparoscopic surgical management of FW hernias specifically is sparse. This necessitated an initial open approach and perhaps sheds light on the need for further guidance on how to manage these cases laparoscopically with the incidence of internal hernias increasing in practice. 

## Conclusions

Internal hernias, especially through the FW, are a diagnostic challenge due to their rarity and the non-specificity of symptoms. In this case, the diagnostic challenge presented in the form of non-specific symptoms and a prior diagnosis attributed to her symptoms. With the availability of cross-sectional imaging, a diagnosis was reached swiftly when new information was available. In terms of surgical management, with a limited number of cases of FW hernias in the literature, experience or guidance in laparoscopic surgical management is sparse. This necessitated an initial open approach to our case and perhaps sheds light on the need for further guidance on how to manage these cases laparoscopically with the incidence of internal hernias increasing in practice.
